# The difference in cortical activation pattern for complex motor skills: A functional near- infrared spectroscopy study

**DOI:** 10.1038/s41598-019-50644-9

**Published:** 2019-10-01

**Authors:** Seung Hyun Lee, Sang Hyeon Jin, Jinung An

**Affiliations:** Convergence Research Center for Wellness, DGIST, Daegu, Republic of Korea

**Keywords:** Motor cortex, Near-infrared spectroscopy

## Abstract

The human brain is lateralized to dominant or non-dominant hemispheres, and controlled through large-scale neural networks between correlated cortical regions. Recently, many neuroimaging studies have been conducted to examine the origin of brain lateralization, but this is still unclear. In this study, we examined the differences in brain activation in subjects according to dominant and non-dominant hands while using chopsticks. Fifteen healthy right-handed subjects were recruited to perform tasks which included transferring almonds using stainless steel chopsticks. Functional near-infrared spectroscopy (fNIRS) was used to acquire the hemodynamic response over the primary sensory-motor cortex (SM1), premotor area (PMC), supplementary motor area (SMA), and frontal cortex. We measured the concentrations of oxy-hemoglobin and deoxy-hemoglobin induced during the use of chopsticks with dominant and non-dominant hands. While using the dominant hand, brain activation was observed on the contralateral side. While using the non-dominant hand, brain activation was observed on the ipsilateral side as well as the contralateral side. These results demonstrate dominance and functional asymmetry of the cerebral hemisphere.

## Introduction

Human motor and sensory functions are controlled by closely related neural networks, and the cortical and subcortical structures of each neural network are involved in processing various pieces of information. Lesion studies on the functional areas of these human motor systems have relied on a variety of techniques and the anatomy and physiology of patients with brain lesions^1,2^. To date, most studies on motor function have mainly mapped the brain activation patterns during exercise^3–5^. These studies have generally reported that complex motions lead to activation of motor-associated regions and activation of the primary motor and sensory regions^6^. However, in addition to cortical activity, the asymmetry of the cerebral hemisphere and dominant hand are inevitable in human motor function. Previous studies have found significant differences in the activation patterns of right and left hand movements^7^. Other studies have reported that the left brain is simply repetitive, and that the activity of the right brain is lateralized in tracking tasks that require visual cortex coordination^8^. In this way, the motor functions of the dominant and non-dominant hands are different, and functionally related cranial nerves are used.

Previously, functional magnetic resonance imaging (fMRI) was widely used as a method for studying functional brain activity. However, since fMRI poses a large limitation on subject movement, such studies have been performed mainly on finger movements that are relatively easy to perform^9,10^. It is rather difficult to evaluate brain function by means of the same tools used during training. Although there is a functional difference between the dominant and non-dominant hemispheres, there are few studies on the effects of complex motions on brain activation patterns. In this study, chopsticks were used for complex exercise. The use of chopsticks required fine motor ability of the hand, gross motor ability of the arm, attention, and eye-hand coordination. Use of chopsticks is thus a suitable means of performing complicated hand movements. fNIRS was used to assess brain activation. fNIRS is a non-invasive brain imaging technique based on the principle of neurovascular coupling^11,12^. Neurovascular coupling indicates the relationship between neural activity and changes in cerebral blood flow^13^. Neural activity causes changes in cerebral hemodynamics leading to blood flow to the activated brain area^14,15^. The local oxygen supply is greater than the oxygen consumed, so higher concentrations of oxygenated hemoglobin (HbO) and lower concentrations of deoxygenated hemoglobin (HbR) are observed in the activated brain region^16,17^. The principle of measurement was developed by Jobsis^18^, based on the measurements of hemoglobin oxygenation in the cerebral blood. A portable, non-invasive, and inexpensive method for monitoring cerebral hemodynamic activity using near-infrared light^19^, fNIRS measures the relative changes in the concentration of HbO and HbR, and it has a relatively high temporal resolution and robustness for motion compared with fMRI. Due to these advantages, fNIRS is widely used in studying rehabilitation, activated brain areas, and underlying mechanism, as well as brain plasticity. In this study, we attempted to compare the difference between the dominant and non-dominant cerebral hemisphere based on the brain activation pattern during the performance of complex tasks.

## Results

### Results of brain activation

**Figure 1:**
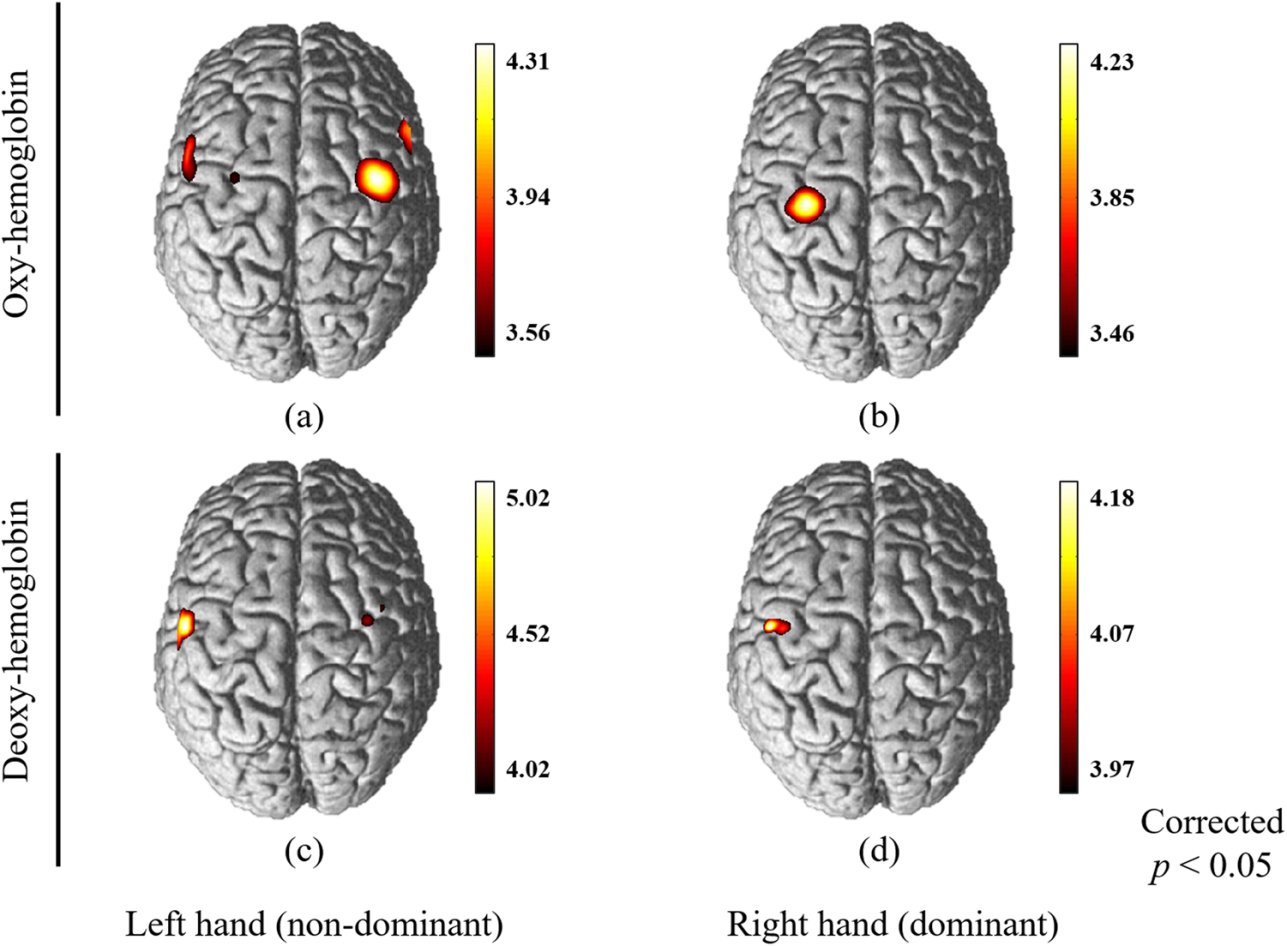
Cortical activation patterns of chopstick tasks. (**a**) cortical mapping based on changes in oxy-hemoglobin using left hand (non-dominant), (**b**) cortical mapping based on changes in oxy-hemoglobin using right hand (dominant) movement. (**c**) cortical mapping based on changes in oxy-hemoglobin using left hand (non-dominant) and (**d**) cortical mapping based on changes in oxy-hemoglobin using right hand (dominant) movement.

Figure 1 shows the cortical activation maps in terms of HbO and HbR in response to the use of chopsticks with the left and right hands. The cortical activation maps showed statistically significant differences between left and right hand movements. In the group analysis of HbO (Fig. 1[a],[b]), use of the right hand (dominant) led to activation of the left primary motor area (M1), whereas use of the left hand (non-dominant) led to activation of the bilateral motor cortex, and premotor area. In the group analysis of HbR (Figure [c],[d]), when using the right hand (dominant), activity was also observed in the left M1. When using the left hand (non-dominant), activation was observed in the bilateral M1. In the analyses of both HbO and HbR, the use of both hands showed similar brain activity: mainly active in the athletic area, and less active in the full and supplemental areas. When using the right hand (dominant), unilateral activity tendency appeared, whereas when using the left hand (non-dominant), both sides showed activity tendency.

### Time-series analysis

**Figure 2:**
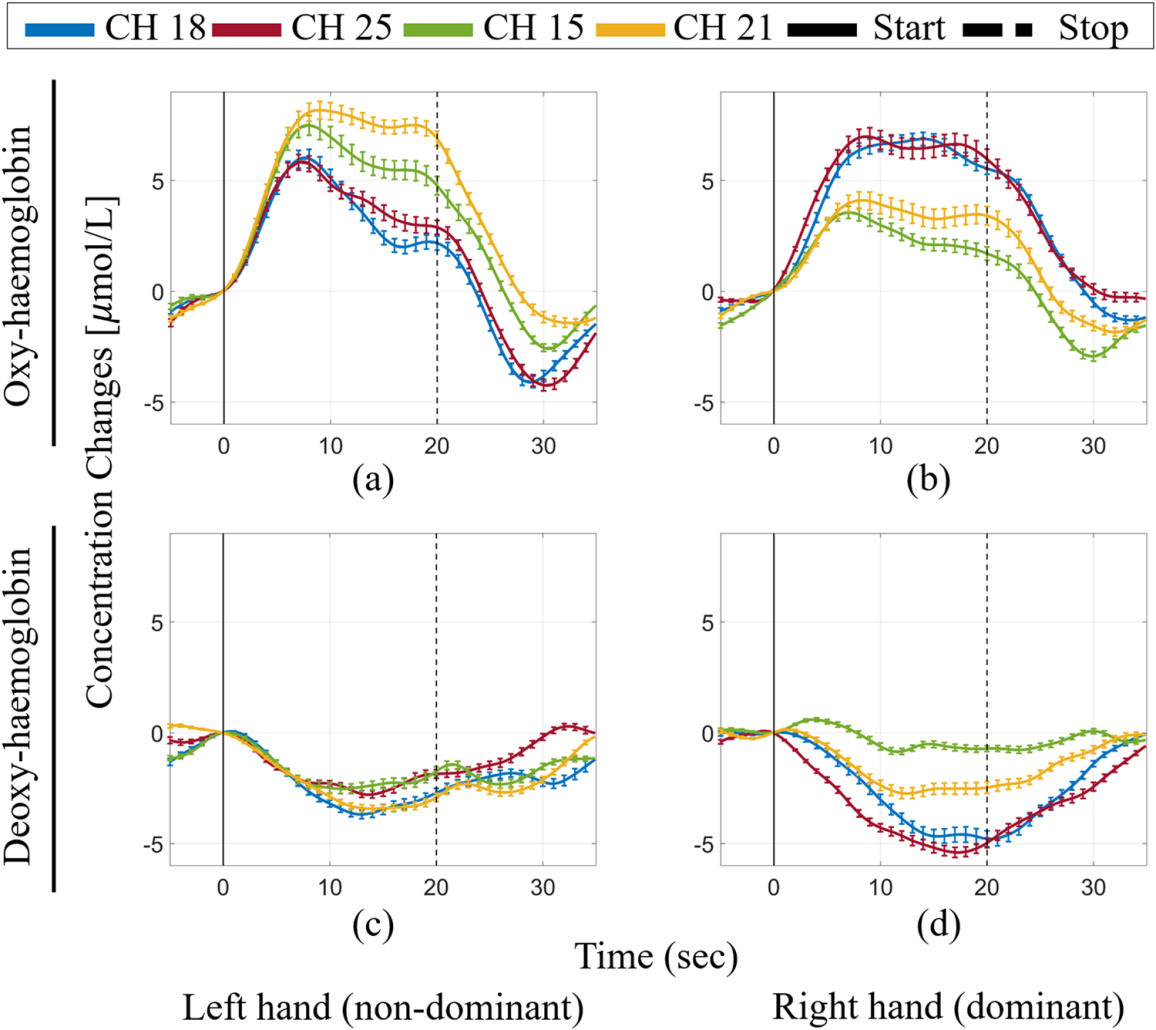
Representative plots of changes in oxy- and deoxy- hemoglobin concentration in the M1. (**a**) changes in oxy-hemoglobin during left hand (non-dominant) and (**b**) right hand (dominant) movement. (**c**) changes in deoxy-hemoglobin during left hand (non-dominant) and (**d**) right hand (dominant) movement.

The average group (n = 15) changes in HbO and HbR concentrations in the primary motor cortex (M1) during each motor task are compared in Fig. 2. We focused on the right and left M1 because they are significant regions in tasks. We thus selected channels 15, 18, 21, and 25 as the regions of interest (ROIs). Channels 18 (blue line) and 25 (red line) represent the left hemisphere, while channels 15 (green line) and 21 (yellow line) represent the right hemisphere. Black lines indicate the task start (solid line) and task end (dashed line). When using the right hand (dominant), there was a significant increase in HbO in the M1 of the left (contralateral) hemisphere compared with the M1 of the right (ipsilateral) hemisphere (Fig. 2b), whereas when using the left hand (non-dominant), concentration changes in blood were significantly increased in the left and right M1 areas (Fig. 2a). With respect to the HbR signal, when using the right hand (dominant), there was a significant decrease in the left hemisphere M1 compared with the right hemisphere (Fig. 2d) and a similar decrease in the left and right sides when using the left hand (non-dominant) (Fig. 2c). Comparison of the mean values of HbO and HbR was performed to test the existence of group activation. The mean values of the concentration changes in HbO and HbR (averaged from 5 s to 15 s after the task) were calculated for each task block. The mean values of HbO and HbR were compared between the left and right hemispheres using the two sample *t*-test to evaluate whether they were significantly different. The mean value of HbO was significantly higher in the left hemisphere than in the right hemisphere for right hand movements (left hemisphere: 0.0064 ± 0.00005, right hemisphere: 0.0033 ± 0.00005, *p* = 0.013). The HbR response in the left hemisphere was also significantly lower than that in the right hemisphere for right hand movements (left hemisphere: −0.0035 ± 0.00003, right hemisphere: −0.0012 ± 0.00002, *p* = 0.005). However, when using the left hand, there was no significant difference in the activity between the left and right hemispheres in terms of both HbO and HbR (right hemisphere: 0.0071 ± 0.00007, left hemisphere: 0.0047 ± 0.00006, *p* = 0.086 for HbO, right hemisphere: −0.0025 ± 0.00002, left hemisphere: −0.0026 ± 0.00002, *p* = 0.828 for HbR). Table 1 shows the mean activation in each brain region for right and left hand movements.

**Table 1:** Average activation in brain regions during use of chopsticks with dominant and non-dominant hands.

Hand	Hemoglobin	Region		*t*-value	*p*-value
		Left hemisphere	Right hemisphere		
Left hand (non-dominant)	HbO	0.0047 ± 0.00006	0.0071 ± 0.00007	−1.729	0.086
	HbR	−0.0026 ± 0.00002	−0.0025 ± 0.00002	−0.216	0.828
Right hand (dominant)	HbO	0.0064 ± 0.00005	0.0033 ± 0.00005	2.513	0.013*
	HbR	−0.0035 ± 0.00003	−0.0012 ± 0.00002	−2.816	0.005**

Values are mean ± standard deviation.

*p < 0.05, **p < 0.01

HbO: oxygenated hemoglobin, HbR: deoxygenated hemoglobin.

### Results of Lateralization

**Figure 3:**
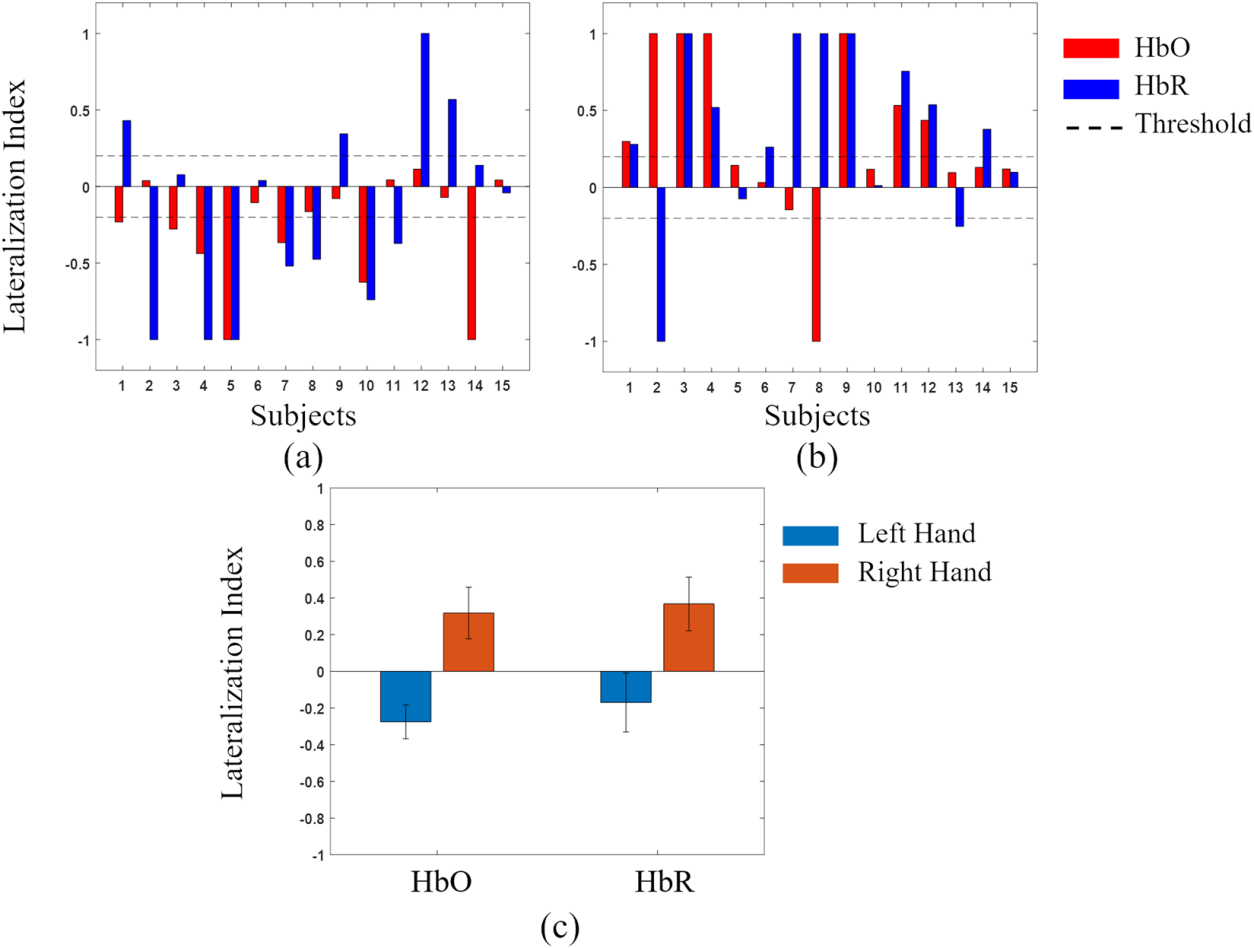
Laterality values for the right and left hands while using the chopsticks. (**a**) individual laterality index for left hand using the chopsticks, (**b**) individual laterality index for right hand using chopsticks, (**c**) average values of laterality index for the right and left hands. HbO: oxygenated hemoglobin; HbR: deoxygenated hemoglobin.

The lateralization index (LI) used the *t*-value of the M1 channel of the left and right hemispheres. In this study, the value of LI threshold (LI_TH_) was set to 0.2. In the individual LI, when using the right hand (Fig. 3[b]), in 7 of 15 subjects, dominance was found in the left hemisphere based on HbO, and in 10 of 15 subjects, dominance was found on the left hemisphere based on HbR. When the left hand was used, dominance was found on the right side in 7 of 15 subjects based on HbO and HbR. The mean LI value (for both HbO and HbR) was unilaterally stronger when using the right hand (dominant) than when using the left hand. Figure 3(c) shows the bar graphs of the average LI of the left and right hands during hemispheric lateralization. The Y-axis indicates the average LI, and the error bars indicate the standard errors. The mean LI values were 0.3176 ± 0.525 and −0.2749 ± 0.344 in the right and left hands for HbO, respectively, and 0.367 ± 0.545 and −0.1702 ± 0.600 in the right and left hands for HbR, respectively (Table 2).

**Table 2:** Statistical results of lateralization index in HbO and HbR.

Hemoglobin	Hand (Lateralization index)		*t*-value	*p*-value
	Left hand	Right hand		
HbO	−0.2749 ± 0.344	0.3176 ± 0.525	−3.526	0.0014**
HbR	−0.1702 ± 0.600	0.367 ± 0.545	−2.481	0.0194*

Values are mean ± standard deviation.

*p < 0.05, **p < 0.01.

HbO: oxygenated hemoglobin, HbR: deoxygenated hemoglobin.

## Discussion

This study was designed to observe brain activation patterns in related motor areas when complicated movements (the use of chopsticks) were performed with dominant and non-dominant hands. The use of chopsticks is a complicated action involving fine (finger), coarse (arm), and sequential (holding, moving, and releasing) movements, and fNIRS can measure corresponding changes in cerebral blood volume. Using the brain mapping method, we analyzed brain activation patterns by obtaining fNIRS signals while the subjects performed the chopsticks task using both hands. There was a significant difference in brain activity during the use of the dominant and non-dominant hands while performing the chopsticks task. When the left hand (non-dominant) was used, high brain activity was detected on the contralateral as well as the ipsilateral side, showing a bilateral tendency.

The existing studies on cerebral cortical activity during motor functions can be divided into two groups: studies comparing activation patterns of the cerebral cortex during simple repetitive tasks and studies on brain activation patterns during sequential exercise^20–22^. For simple repetitive motions, fNIRS signals were obtained during the clenching of both hands^23–25^. These results showed that fNIRS signals were unilaterally localized on the right (dominant) and left (non-dominant) sides. On the other hand, while performing complicated movements (sequential finger movements), brain activation was observed in the M1 on both sides and in the primary sensory-motor cortex (SM1) in the ipsilateral hemisphere, showing a tendency of bi-laterality^21,26,27^. This study also confirms that there is a difference between the use of dominant and non-dominant hands while performing complex tasks. When the dominant hand was used, high levels of activity were observed in the contralateral M1, S1, PMC, and SMA. When the non-dominant hand was used, activity was observed in M1, S1, both SMA sides, and the contralateral side. Unlike the results obtained for simple repetitive motions, the SMA and PMC showed high activity when the dominant hand was used. This activation was associated with the self-paced movements, and also with preparation, sequencing, and coordinated movements of the hands^28–30^. The abundant connectivity of motor related areas contributes to more complex movements, that is, the coordination of both hands^20^. As secondary motor areas, the PMC and SMA are involved in the planning, preparation, and initiation of movement^31,32^. The SMA is also involved in the internal generation of sequential motor tasks^33–37^. Since the use of chopsticks involves visually guided movement, SMA activation was observed while using chopsticks.

Therefore, since the chopstick task in this study included not only sequential operations but also coarse and fine operations, the activity of SMA seems to be required for movement of both the dominant and non-dominant hands. However, differences in unilateral and bilateral brain activities were more prominent in the M1 area. There was a statistically significant difference in the activity between contra- and ipsi-hemispheres during the use of chopsticks with the dominant hand. Meanwhile, there was no statistically significant difference in the activity of bilateral hemispheres during use of the non-dominant hand. This phenomenon has been reported in previous studies^38,39^. Another study reported connectivity between both the M1 regions^39^. The difference between the dominant and non-dominant hands is also reflected in the LI of the M1 region. During the task, the results for both hands tended to be lateralized on the contralateral side, but when using the non-dominant hand, high brain activity was observed in the ipsilateral M1, and the lateralization of brain activity was reduced (HbO; Lt: −0.2749, Rt: 0.3176, HbR; Lt: −0.1702, Rt: 0.367). These results for the M1 region seem to be more dependent on the complex task than those for the other regions. These areas may play a fundamentally different role in the regulation of exercise.

For the dominant and non-dominant hands, anatomical or functional differences exist in the cranial nerve circuit, which is described as hemispheric dominant and functionally asymmetric for the left and right cerebral hemispheres. In a study of motor evoked potentials using transcranial direct current stimulation for left- and right- handed persons, the threshold of right- handed individuals was lower than that of left-handed individuals. This result indicates the difference in the right and left motor nerve pathways^40^. In addition, in a study on differences in brain activity during repetitive opposing movements of the right and left hands, assessed using fMRI, during repetitive opposing movements of the right and left hands, activation was observed in the right hemisphere for right handed individuals, while activation of both hemispheres was observed in left handed individuals^41^. As reported in previous studies, the results of this study showed unilateral changes in brain activation due to right hand movements. During left hand movements, both hemispheres were activated even though they showed a tendency to laterality. This may be due to functional differences in the left and right hands and functional asymmetry of the dominant and non-dominant hemispheres. In addition, in a study on motor learning through repetitive training, activity in the frontal lobe, the PMC, and SMA, including the SM1, in the early stage of motor learning tended to increase but then decreased in the later period^42–44^. This shows that the degree of learning depends on the area of the brain used to perform the task, and the efficiency of the neuronal activity for the non-dominant hand is different from that of the dominant hand. Since the subjects in this study had used chopsticks with their right hands for a long time, these results suggest a functional difference between the dominant and non-dominant hemispheres due to genetic and environmental influences. In this study, we attempted to observe the differences in brain activity when performing complex tasks using the dominant and non-dominant hands, and we conducted experiments related to the laterality of brain function during the task, using fNIRS. These experiments are expected to serve as a basis for measuring brain activity according to various quantifiable exercise tasks in the future. However, it is necessary to statistically generalize the difference in brain activity during exercise between the dominant and non-dominant hemispheres to more subjects. It is expected that changes in the cerebral cortex due to non-dominant learning will be studied in various areas.

The purpose of this study was to investigate the brain activation patterns during a complicated task involving the dominant and non-dominant hands. For this purpose, we recruited fifteen healthy participants and analyzed hemodynamics responses obtained during the chopstick tasks. One of the main goals of this study was to determine the asymmetry of the cerebral hemisphere using fNIRS. Only right-handed subjects participated in this study. Experiments on left-handed subjects should be performed in the future to confirm the asymmetry of the cerebral hemisphere. It is also important in investigating motor learning to analyze cerebral functions. Motor learning is a complex process of the brain that changes the central nervous system and allowing for the production of new motor functions. Experiments need to be conducted in the future to prove motor learning processes. Despite these limitations, these results are meaningful and helpful for rehabilitation because we have demonstrated the feasibility of using fNIRS for asymmetry of the cerebral hemisphere during motor tasks. These results revealed activity in the M1 for both dominant and non-dominant hand tasks. During dominant hand movements, activity was lateralized on the contralateral side. In contrast, during the non-dominant hand movement, activity was in the ipsilateral as well as in the contralateral side. This suggests a difference between the left and right cerebral hemispheres due to dominant and functional symmetry.

## Materials and Methods

The study was approved by the by the Institutional Review Board of Daegu Gyeongbuk Institute of Science & Technology (DGIST-170816-HR-030-01) and performed according to the Declaration of Helsinki. The methods used in this study were performed in accordance with the guidelines approved by Institutional Review Board of the Daegu Gyeongbuk Institute of Science & Technology.

### Subjects and experimental design

Fifteen healthy right-handed subjects (5 men, 10 women; mean age 27.4 years, range 18~36 years) were included in this study. The Edinburg Handedness Inventory^45^ was used for the evaluation of handedness. All subjects understood the purpose of the study and gave informed written consent prior to the experiments. Subjects with a history of neurological or physical illness or those who could not hold chopsticks correctly were excluded from this study.

**Figure 4:**
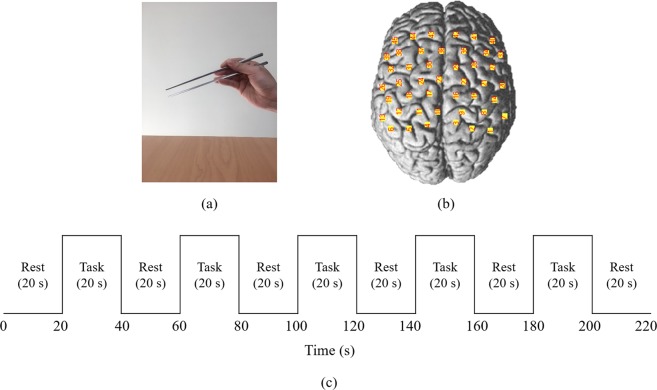
Experimental setup and protocol. (**a**) hand position with chopsticks, (**b**) configuration of fNIRS channels, and (**c**) chopsticks protocol used in the experiment.

Two motor tasks (using chopsticks with the right and left hands) were performed in this study. The subjects were asked to sit comfortably in a chair in an upright position during the experiment. Two containers were placed on the desk in front of the subjects. The experimental tasks included transferring the almonds from the left container to the right one with the right hand and from the right container to the left one with the left hand. When using stainless steel, the subjects were asked to use the chopsticks right way (Fig. 4[a]). The subjects were instructed to practice two motor tasks several times before the experiments. The experiments were arranged in a block paradigm. The block design consisted of 6 rest and 5 task periods, each of a 20 s duration, with alternating rest and task periods (Fig. 4[c]). The two motor tasks were performed twice with a self-paced frequency, and the sequence of the tasks was assigned randomly.

### fNIRS measurement

A commercial continuous-wave fNIRS system (FOIRE-3000; Shimadzu, Japan) was used to measure the time course of HbO and HbR in this study. This system performed near-infrared topographic measurements at three different wavelengths (780, 805, and 830 nm) at a sampling rate of 10 Hz. In order to include the bilateral sensorimotor areas and allow group analysis across subjects, the optodes were positioned based on the 10–20 international electrode system. The system included 15 sources and 15 detectors, with a 30-mm spatial separation between sources and detectors. The optode configuration resulted in 45 channels that included the SM1, PMC, SMA, and frontal cortex (Fig. 4[b]). The midline of channels 16 and 17 (i.e., transmitter 6) was placed in Cz. Registration of optode locations was performed using a three-dimensional position measuring system (FASTRAK, Polhemus, Colchester, VT, USA). Subsequently, Montreal Neurological Institute coordinates of each channel were estimated using NFRI fNIRS tools^46^, and the correspondence of the fNIRS channels to the underlying cortical areas was estimated based on a virtual registration method without MRI^47^ using the SPM software^48^. The coordinates of the ROIs were shown in Table 3.

**Table 3:** Localization of fNIRS channels, MNI coordinates and Brodmann areas.

Hemisphere	Channel	MNI coordinates			BA (%)
		x	y	z	
Left hemisphere	18	−35	−20	73	Primary Motor Cortex (53%) Pre-Motor and Supplementary Motor Cortex (47%)
	25	−44	−8	63	Pre-Motor and Supplementary Motor Cortex (66%) Primary Motor Cortex (34%)
Right hemisphere	15	30	−20	75	Pre-Motor and Supplementary Motor Cortex (68%) Primary Motor Cortex (32%)
	21	41	−8	67	Pre-Motor and Supplementary Motor Cortex (75%) Primary Motor Cortex (25%)

MNI: Montreal neurological institute, BA: Brodmann areas.

### Data processing and analysis

#### Brain Mapping

The analysis was performed using the open source software package NIRS-SPM implemented in MATLAB (MathWorks, Inc., Natick, MA, USA). The concentration changes in HbO and HbR for each channel were obtained using the modified Beer–Lambert law^49^. The time-series of the hemoglobin concentration was subsequently subjected to independent component analysis (ICA) to remove typical motion artifacts^50,51^. The hemodynamic response function was applied for correction of noise from the fNIRS system, and the wavelet-MDL based detrending algorithm^52^ was used for correction of low frequency drift. The GLM for analysis of fNIRS data has been well established^53,54^. The general linear model (GLM) analysis with canonical hemodynamic response curve was performed to model the hypothesized HbO and HbR response and to test for significant cortical activation during experimental conditions^55,56^. At the group level, statistical analysis was performed based on the individual-level beta-values to find activated channels (*p* < 0.05, FDR-corrected)^57^. Furthermore, *t*-statistic maps computed for group analysis were plotted onto a conventional brain template, and the regions with significant differences in HbO and HbR concentrations were identified.

#### Time-series analysis

The M1 region related to hand movement was selected as the ROI, and the activities of both hands were compared. The concentration changes of HbO and HbR were estimated from each channel of the ROI during each motor task. For each channel, the grand average of each hemodynamic response was computed. For comparison of the activities of the bilateral M1 regions, the mean values of the concentration changes of HbO and HbR were obtained from the averages of the ROI channels (left hemisphere M1: channel 18 and 25, right hemisphere M1: channel 15 and 21). The hemodynamic response is delayed about 5 s from the onset of neural activity, after which it gradually returns to baseline. Taking into account the hemodynamic response, we chose a time window of 5 s to 15 s after the task onset. This window was chosen to include the maximum variations of HbO and HbR concentrations. Data from a total of 75 blocks were used across the 15 subjects (total 75 blocks, 5 blocks × 15 subjects). Two-sample *t*-test was used for determination of the significance of the differences in hemodynamic responses in the left and right hemisphere during two motor tasks (using chopsticks in the left hand and right hand).

#### Lateralization Index

Through GLM analysis for fNIRS we could calculate the *t*-value of each channel. The *t*-value obtained from the statistical analysis indicated activation of the cerebral cortex. Thus, we used the *t*-values to analyze the symmetry/asymmetry in the activity of the primary motor cortex in response to the use of chopsticks with the left and right hands. LI was calculated for *t*-values of ROI channels using the formula (LH_*t*-value_ − RH_*t*-value_)/(LH_*t*-value_ + RH_*t*-value_), where LH_*t*-value_ and RH_*t*-value_ indicated *t*-value of the left (channel 25) and right (channel 21) hemispheres during the tasks, respectively^58^. Hemispheric dominance is typically determined by comparing LI to a predefined threshold (LI_TH_), according to the following rule:

1 $$\begin{array}{c}if\,LI > L{I}_{TH}:left\,hemispheric\,dominance,\,\\ else\,if\,LI < -\,L{I}_{TH}:right\,hemispheric\,dominance,\,\\ else\,if\,|LI|\le L{I}_{TH}:bilateral\,dominance\end{array}$$

In this study, the threshold LI_TH_ value is set to 0.2^59,60^.

### Ethical approval and informed consent

The study protocol was approved by the Institutional Review Board of the DGIST (DGIST-171221-HR-039-02), and all participants provided written informed consent.
